# Experts’ opinion about the primary headache diagnostic criteria of the ICHD-3rd edition beta in children and adolescents

**DOI:** 10.1186/s10194-017-0818-y

**Published:** 2017-11-23

**Authors:** Aynur Özge, Noemi Faedda, Ishaq Abu-Arafeh, Amy A. Gelfand, Peter James Goadsby, Jean Christophe Cuvellier, Massimiliano Valeriani, Alexey Sergeev, Karen Barlow, Derya Uludüz, Osman Özgür Yalın, Richard B. Lipton, Alan Rapoport, Vincenzo Guidetti

**Affiliations:** 1 Department of Neurology, Mersin University Medical Faculty, Mersin, Turkey; 2 Phd program in Behavioural Neuroscience, Department of Paediatrics and Child and Adolescent Neuropsychiatry, Sapienza University of Rome, Rome, Italy; 3 Royal Hospital for Sick Children, Glasgow, G3 8SJ UK; 4 UCSF Headache Center and UCSF Benioff Children’s Hospital, Pediatric Brain Center 2330 Post St 6th Floor San Francisco, Campus Box 1675, San Francisco, CA 94115 USA; 5 NIHR-Wellcome Trust King’s Clinical Research Facility, King’s College London, London, England; 6 Division of Paediatric Neurology, Department of Paediatrics, Lille Faculty of Medicine and Children’s Hospital, Lille, France; 7 Division of Neurology, Ospedale Pediatrico Bambino Gesù, Piazza Sant’Onofrio 4, 00165 Rome, Italy; 8 Center for Sensory-Motor Interaction Aalborg University, Aalborg, Denmark; 9 Department of Neurology and Clinical Neurophysiology, University Headache Clinic, Moscow State Medical University, Moscow, Russia; 10 Faculty of Medicine, University of Calgary, Alberta Children’s Hospital, C4-335, 2888 Shaganappi Trail NW, Calgary, AB T3B 6A8 Canada; 11 Cerrahpaşa Medical Faculty, Department of Neurology, İstanbul University, Kocamustafapaşa, İstanbul, Turkey; 12 İstanbul Research and Education Hospital, Kocamustafapaşa, İstanbul, Turkey; 13 Department of Neurology Montefiore Headache Center, Albert Einstein College of Medicine, Louis and Dora Rousso Building, 1165 Morris Park Avenue, Room 332, Bronx, NY 10461 USA; 14 The David Geffen School of Medicine at UCLA, Los Angeles, CA USA; 15 Department of Pediatrics and Child and Adolescent Neuropsychiatry, Sapienza University, Rome, Italy

**Keywords:** Headache, Classification, Childhood headache, Adolescent headache, Primary headache disorders, Migraine, Tension-type headache, Cluster headache

## Abstract

**Background:**

The 2013 International Classification of Headache Disorders-3 (ICHD-3) was published in a beta version to allow the clinicians to confirm the validity of the criteria or to suggest improvements based on field studies. The aim of this work was to review the Primary Headache Disorders Section of ICHD-3 beta data on children and adolescents (age 0-18 years), and to suggest changes, additions, and amendments.

**Methods:**

Several experts in childhood headache across the world applied different aspects of ICHD-3 beta in their normal clinical practice. Based on their personal experience and the literature available on pediatric headache, they made observations and proposed suggestions for the primary headache disorders section of ICHD-3 beta data on children and adolescents.

**Results:**

Some headache disorders in children have specific features which are different from those seen in adults and which should be acknowledged and considered. Some features in children were found to be age-dependent: clinical characteristics, risks factors and etiologies have a strong bio psycho-social basis in children and adolescents making primary headache disorders in children distinct from those in adults.

**Conclusions:**

Several recommendations are presented in order to make ICHD-3 more appropriate for use with children.

## Background

Headache is a frequent cause of pain and of significant disability in children and adolescents. Its varying presentations, etiologies, triggers and methods of management can pose diagnostic and therapeutic dilemmas. Primary headache disorders in childhood are different from their adult manifestations, but the cause for this difference is unknown. It could be the result of differences in degree of brain maturation including myelination, new synapse formation and synaptic reorganization [1, 2].

Owing to the high prevalence of childhood headache and the absence of specific objective diagnostic criteria for children, accurate clinical diagnostic criteria are needed.

## Review

### Methodology

The aim of this study was to receive comments and suggestions from pediatric headache specialists around the world about the accuracies and deficiencies of ICHD-3 beta, and to provide a summary of them. The authors who agreed to be a part of this project have been included in the study team. Most authors belonged to one part of the classification depending on their specialty. Each of them was asked to search the literature written prior to and after the publication of ICHD-3 beta, and to provide their comments and suggestions. The primary source of literature was Pubmed, and the paper also benefited from other widely used search engines, such as Google Scholar, and reference lists from single articles, reviews and editorials. The entire list of publications utilized in this process has been submitted in the reference section of this paper.

## Migraine

### Comments

Migraine in children differs from migraine in adults with regard to its duration, location, and sometimes method of diagnosis, such as inferring symptoms from behavior. A significant body of literature has revealed high prevalence of migraine in the pediatric age group [3–7]. Up to age 7, migraine prevalence of both genders is almost equal, and after age 12, there is a strong female predominance, which increases with age. Between ages 7 and 12, there are no gender-related differentials [8].

Gender influences the manifestation of some migraine-related symptoms. In females, a decrease in physical activity and the presence of aura seem to be more common, whereas in males vomiting and phonophobia occur more frequently [9]. Overall, the estimated reported prevalence of migraine in the pediatric age group (3 to 18 years old) ranges from 7.7% to 17.8%, with a difference of 3.7% between the sexes (9.7% in females vs 6.0% in males [10–12].

The clinical presentation of infants with migraine can be non-specific and the symptoms may be vague. The classical presentation is facial pallor, occasional vomiting, loss of appetite, intolerance to light or noise and irritability [13].

### Migraine without aura

#### Comments

Attacks of migraine without aura in children are often shorter than in adults and bilateral in location. The pain becomes unilateral only in late-adolescence or early adulthood. In children, the most common location of pain is frontal (60.9%), whereas it is ocular in adults (53.17%), followed by temporal (38.67%) [2]. The quality of pain in children is usually throbbing or pounding, whereas in adults it is a frequent pulsating [14].

Younger children may have difficulty describing the quality and severity of their pain, so using a face scale, numerical scale, or even asking them to draw a picture, can be extremely helpful in determining the characteristics [15]. In some otherwise typical patients, the pain is localized to the face, but there is no evidence that these patients form a separate subgroup. Also, because young children may not be able to describe their pain, sensitivity symptoms such as photophobia and phonophobia may be inferred from their behavior, i.e. avoidance of these stimuli. Migraine attacks may be associated with cranial autonomic symptoms and cutaneous allodynia [16]. Migraine without aura peaks at ages 14-17 years in girls and 10-11 years in boys [10–12, 17–20].

According to the ICHD-3 beta, occipital headache in children is rare and should be diagnosed with caution. Some studies from emergency departments specifically identified occipital headache in children relating to neurologic findings as elevated intracranial pressure, tumors, benign cysts, or sinusitis on imaging [21–24].

In very young children, the diagnosis of migraine can be particularly challenging due to their inability to verbalize symptoms, such as photophobia or phonophobia. Reports are commonly based on parent or care-taker’s observations, which are often at the core of diagnosis. The proposed diagnostic criteria for migraine in younger children, which emphasize behavioral cues rather than verbal reports, are summarized in Table 1, as proposed by Mc Abeee et al. before and modified by the current authors [3].

**Table 1:** Proposed criteria for migraine in children age 5 years and younger

A. At least five headache attacks fulfilling the criteria from B to D
B. The headache lasts for 30 min or longer (untreated or treated)
C. The headache has at least one of the following characteristics:
1. Pain of at least moderate severity
2. Unilateral or bilateral headache
3. Throbbing or pounding nature of pain,
4. Exertion intolerance; avoidance of walking or playing;
D. The headache is associated with at least one of the following:
1. Loss of appetite, stomach discomfort or dizziness
2. Sensitivity to light and sounds as indicated by inability to watch TV or play on the computer or on electronic games
3. Having one cranial autonomic symptom associated with headache attacks
E. Not better accounted for by other diagnoses

#### Recommendations

1. Recognition of the short duration of migraine attacks should be emphasized and highlighted.
2. Frontal Headache is common in children with migraine without aura and should be acknowledged.
3. Severity of headache is best described by the effect of headache on daily activities. Mild if no interference, severe if it stops all activities, moderate if it interferes with some, but not all activities.
4. Presence or absence of light and noise intolerance can be inferred from change in behavior and reactions to light and noise, rather than by asking a direct question.
5. In a child with a normal neurologic examination and a headache phenotype consistent with migraine, an occipital location of the head pain without any additional red flag does not seem to increase the likelihood of a secondary cause.
6. It should be emphasized that the character of pediatric migraine changes over time. The distinction between migraine and tension-type headache is far less sharp in children than adults. Additional criteria may be needed, such as presence of motion sickness or osmophobia.
7. Vertiginous symptoms and motion sickness seem to be supporting features of pediatric migraine.
8. A sense of aural fullness should be added as a cranial autonomic symptom of pediatric migraine.

### Migraine with aura

#### Comments

In children and adolescents with migraine, aura is usually, but not always, unilateral [25]. Visual auras were the most common occurrence (87.1%) in children and adolescents as well as in adults. Adolescents were 3 times more likely to experience aura compared to pre-pubertal children, but it is possible this different prevalence of aura in patients of different ages can be explained by children’s ability to describe their symptoms [26].

A validated, highly specific and sensitive analogue scale is available for rating visual aura [27].

Second in frequency are sensory disturbances in the form of pins and needles migrating slowly from the point of origin (often the hand or face) and affecting a greater or smaller part of one side of the body, face, and/or tongue. Numbness may occur in their wake, but numbness may also be the only symptom. Less common are speech disturbances, usually aphasic but often hard to categorize. Prognostically, 50% to 60% of adolescents with migraine with aura are still symptomatic at the 5- to 7-year follow-up [10–12, 18–20, 28–30]).

#### Associated autonomic features

In pediatric migraine, the presence of cranial autonomic symptoms appears to be the rule rather than the exception (about 62% of cases). The data of Gelfand et al. [28] showed that approximately two-thirds of patients in pediatric/adolescent neurology or specialty headache practices with migraine have accompanying cranial autonomic symptom. By contrast to adults, they are usually bilateral. Clinicians should be careful to consider migraine when evaluating a child with headache and associated ocular or nasal symptoms in order to avoid a misdiagnosis of sinus headache (nearly 40% of pediatric migraineurs) [3, 28].

#### Recommendations

The autonomic symptoms associated with children and adolescent migraine should not be considered as exceptions because there are substantiated data that suggest that they can be observed in the presence of reported aura or without aura symptoms, on its own.

### Chronic migraine

#### Comments

Chronic migraine (CM) occurs in 1.7% of children, with an increase in frequency with age (doubled at 12 years) but only 1.5% of adolescents. The significant risk factors are advancing age, sex (favoring girls) and family history of headache (father and sibling). This is an important issue, with or without consideration of medication overuse [11, 12, 19, 20]. One study on 1134 children and adolescents with chronic headache (73.2% with migraine) revealed that migraine equivalents (abdominal migraine, cyclical vomiting, benign paroxysmal vertigo, and benign paroxysmal torticollis) are so common that these should potentially be considered as part of the migraine syndrome. The ICHD-3rd beta version lists these equivalents as separate disorders (1.6–1.6.3).

#### Recommendations

CM is a growing problem of children and adolescents, as is the case with adults [31]. Risk factors and predisposing medical problems should be revealed by physicians.

### Episodic syndromes that may be associated with migraine

#### Comments

There are several episodic syndromes in children and adolescents, discussed often not only in terms of diagnosis, but also the prognostic importance. On the other hand, some symptoms like motion sickness or vertigo, associated with headache disorders, are still debated [32–35]. Among these episodic syndromes, recurrent abdominal pain (RAP), also known as “Abdominal Migraine”, is defined as three or more episodes of abdominal pain which persists for more than 3 months and are severe enough to interfere with normal activities [36]. For most physicians, selecting the appropriate diagnostic modality is difficult. Often, there is a positive family history of migraine and evidence suggesting that RAP is a precursor of migraine headache, and primarily a comorbid pain disorder in preadolescent patients [37].

Benign paroxysmal vertigo of childhood begins between ages 2 and 5 years, with some children outgrowing the disorder and others showing persistent symptoms into adolescence; cyclic vomiting syndrome begins on average at age 5 (about 40% rate of headache); and abdominal migraine usually begins latest, at school-age. Children with periodic syndromes appear healthy, and neurologic findings between attacks are absent. Often, these children have a family history of migraine. Those with the earlier-onset syndromes may have later-onset types of periodic syndromes or migraine headaches later in childhood, and even in adulthood [36, 38–41].

Recent research suggests that infant colic, defined as excessive crying in an otherwise healthy infant, may also be a childhood periodic syndrome. The prevalence rate is 5% to 19%. It usually peaks around 6 weeks of life and resolves by the age of 3 months. There is some supporting evidence linking colic in infancy to migraine headaches in adulthood. Recent studies showed that mothers with migraine are more than twice as likely to have an infant with colic [34, 38–45].

#### Recommendations

1. The following comment should be added: The clinical phenotype of childhood periodic syndromes changes with the developing brain and childhood periodic syndromes may present before migraine headache.
2. The definition of benign paroxysmal torticollis should include recurrent episodes of one-sided head tilt with slight rotation, with onset in the first year.
3. Infant colic should be moved from the appendix to the main body of ICHD-3 in the final version.
4. Familiarity with these disorders and their association with migraine headache may help clinicians reach the correct diagnosis and guide their selection of therapy.

Key point 1: In a child with a normal neurologic examination and a headache phenotype consistent with migraine, an occipital location of the head pain should lead to caution in diagnosis, but a secondary cause may not be found.

Key point 2: In young children the diagnosis of migraine can be particularly challenging due to their inability to verbalize symptoms; so it is essential to pay attention to behavioral clues rather than verbal reports, such as exertion intolerance and avoidance of walking or playing.

Key point 3: The duration of headache attacks in children should be changed to 30 min to 48 h. Reducing the time duration could increase the number of children diagnosed with migraine.

## Tension-type headache

### Comments

Tension-type headache (TTH) has a wide-ranging reported prevalence of 0.9% to 72.3% in children and adolescents [46]. In clinical studies, chronic tension-type headache was found in 5% to 20.5% of children. Mean age at onset of episodic tension-type headache is 7 years. The average frequency of attacks is two per month, and the duration of each episode is about 2 h. In children, tension-type headache may be triggered by psychosocial stress. It may also be associated with, psychiatric disorders, oro-mandibular dysfunction or muscular stress. Anxiety and mood disorders are common comorbidities, especially when the tension-type headache is chronic [3, 47].

Episodic tension-type headache may have particular features in children. Pain generally starts in the afternoon at school and often the child can carry on with favorite activities despite severe or constant headache. As the headaches are often rare or absent during extended holidays, clinical confirmation of the diagnosis may require assessment over these periods. The features of tension-type headache may change from preschool age to adolescence [48, 49].

The red flags that alert the physician to conduct further investigations for tension-type headache include sudden severe unilateral headache, particularly in the absence of a family history of migraine, which warrants computed tomography examination to exclude vascular disorders [47, 48].

### Recommendations

1. The ICHD-3 beta criteria for pediatric headache are limited, because they rely on patient descriptions of the features of headache and any associated symptoms.
2. The overlap of some symptoms between migraine and tension-type headache may make it difficult for clinicians to distinguish between them.

### Migraine vs tension-type headache

The symptoms of tension-type headache may also overlap with migraine and a diagnosis of migraine can change to episodic tension-type headache over time [50–53]. This occurs in nearly half of all patients from childhood to adulthood. Diagnostic changes of TTH or migraine may sometimes occur more rapidly. The overlap between the diagnoses is greater in children than in adults. Indeed, given that migraine in children may be characterized by non-pulsating (not a rule), bilateral pain, most young migraineurs can easily meet the first two criteria of tension-type headache (point C). Studies have shown that 30% of children cannot describe the quality of their pain, and 16% cannot report on photophobia and phonophobia, which may be required by the ICHD for the diagnosis of migraine. Thus in children, behavior is more important than words. The best diagnostic clinical characteristics of migraine are pain of moderate or severe intensity, pain aggravated by physical activity, and pulsating quality. Children avoid bright light and loud noise. Furthermore, the attacks usually start in the morning hours (58.5%) and resolve after a period of sleep (76.7%). In tension-type headache (TTH), by contrast, there is no photophobia, no nausea, and no aggravation after physical activity; the pain is usually of mild or moderate in intensity and non-pulsating. Thus, the most specific features distinguishing migraine from tension-type headache are improvement after sleep, presence of nausea and vomiting, worsening with physical activity and photophobia- phonophobia- or osmophobia [54]. It is only as children move through adolescence that their migraines begin to resemble migraine in adults, owing to the process of cerebral maturation [3, 30].

There are clinical findings that suggest that tension-type headache in childhood may become migraine later in life. This suggests that migraine and tension-type headache may not be distinct entities, but two aspects of the same spectrum of benign headache [55]; alternatively as the brain matures migraine biology is more readily expressed and the conditions are distinct. Moreover, both are characterized by the same neurophysiological abnormalities, such as the reduced habituation of evoked potentials, which may reflect poor diagnostic differentiation of the conditions with current ICHD criteria for tension-type headache [3, 56].

## Trigeminal autonomic cephalalgias

Trigeminal autonomic cephalalgias (TACs) are divided into five different headache syndromes: cluster headache (CH), paroxysmal hemicranias (PH), short-lasting unilateral neuralgiform headache attacks with conjunctival tearing and injection (SUNCT), and short-lasting unilateral headache attacks with cranial autonomic symptoms (SUNA) that moved from the appendix section of ICHD-II. The ICHD-3 has re-classified hemicrania continua (HC) to the TAC category as suggested by the authors. The hallmark of these headache syndromes is the presence of unilateral autonomic manifestations during the headache episode [1, 3].

### Cluster headache

#### Comments

The estimated prevalence of cluster headache in the pediatric population (age 3-18 years) ranges from 0.03% to 0.1%, with a male preponderance (2.5:1). Onset in childhood is very rare, and about 5% to 10% of cases in the general cluster headache population start in adolescence (mean age 11–14 years). A positive family history of cluster headache is found in approximately 10% of pediatric cases compared to 25% for migraine [57, 58].

The clinical features of pediatric-onset cluster headache seem to be similar to the adult-onset type. Cranial autonomic features may be less prominent in children than in adults, although there is little difference by age in the distribution among ocular, nasal, and facial autonomic symptoms. Lacrimation is reportedly the most common symptom of pediatric cluster headache, followed by conjunctival injection and nasal discharge. The frequency of cluster periods seems to be lower in childhood than adulthood [3, 29, 59, 60].

### Recommendations

We propose the following amendment to the ICHD-3 criteria for CH supported by McAbee et al. before [3]: compared to adults, the attacks may be less frequent and of shorter duration in younger children. Restlessness may not be severe and difficult to characterize. Like migraine attacks, observation of the behavior is more important than words, especially in young children.

### Paroxysmal hemicrania

#### Comments

Paroxysmal Hemicrania usually begins in the second or third decade of life, although there are several case reports of onset before age 18 years and up to 68 years. Both episodic and chronic paroxysmal hemicranias have been reported in children. The hallmarks of these headaches are the strict unilateral location of the pain, the brief duration (ICHD-3 notes duration of up to 30 min) and the dramatic response to indomethacin. From the few reports, there are some pediatric features not in conformity with the ICHD-3. Facial paleness rather than facial flushing has been noted in children. The attacks often last longer than the maximum 30 min noted by the ICHD-3 but the duration of the attacks has been reported up to 45 min in very young children. Children with PH, who had less than five attacks per day for more than half of the time, as specified by the ICHD-3, have been reported before. Also pain attacks have been reported as midline in location and response to indomethacin is not always universal (as in adults). Finally, some migraine features, including vomiting, throbbing nature of the pain, photophobia, osmophobia, as well as a family history of migraine, have also been reported in some of the cases; this is not mentioned in the ICHD-3 [3, 61, 62].

## SUNCT

### Comments

The syndrome of short-acting unilateral neuralgiform headache attacks with conjunctival injection and tearing (SUNCT) is characterized by three different types of pain: single stabs, groups of stabs, and a saw-tooth pattern in which repetitive spike-like paroxysms occur without reaching the pain-free baseline between the individual spikes. Pediatric-onset SUNCT is very rare, with only 4 cases (3 idiopathic, 1 secondary) reported in the literature. Patient ages ranged from 5 to 11. In all the affected children, the clinical phenotype of the headache resembled adult-onset SUNCT [63, 64].

### Recommendations

We need more cases of SUNA and Hemicrania Continua in children and adolescents to make good recommendations.

## Other primary headache disorders

### Comments

In children less than 12 years old, diagnoses of primary cough headache, primary exercise headache, primary headache disorders associated with sexual activity, primary thunderclap headache, and hypnic headache should be made only after the exclusion of other causes of headache.

### Primary exercise headache (PEH)

#### Comments

Activity-related headaches can be brought on by the Valsalva maneuver (cough headache) or prolonged exercise (exertional headache). They account for 1-2% of all consultations for adult headache in a general neurology department and are very rare in children. It must be added that the current criteria are too inclusive and will not differentiate between true PEH and migraines provoked by exertion, a limitation that equally applies to children and adults [3, 65, 66].

### Primary headache associated with sexual activity

#### Comments

A case report of a 12-year-old boy described an association between primary exertional headache and primary sex headache. Others found that comorbid migraine occurs in about 50% of individuals with primary sex headache, perhaps explaining why sex headache has migraine features in some cases [67].

#### Recommendations

The following might be added to the text: “There is a phenomenological association between primary exertion headache and migraine attacks”.

### Primary stabbing or “ice-pick” headache

#### Comments

The estimated prevalence of icepick headache in children is 3-5%. It usually appears by age 10. Even in some reports to the contrary, children and adolescents have been reported to have a higher incidence of co-morbid migraine, TTH, and PEH. The ICHD-3 criteria include duration of a few seconds, but some pediatric reports note duration of up to 15 min. An association with migraine is noted by the ICHD-3 beta version. Also, extra-cephalic pain (abdominal, low back, chest, and knee) has been reported as co-morbidity in a child supported by the authors observations [3, 68, 69].

### New daily persistent headache

#### Comments

New daily persistent headache (NDPH) is very common amongst adolescents, but it is commonly unrecognized and under evaluated. The reported pediatric series note two additional issues associated with childhood NDPH. First, many children either have an antecedent infection, especially like Epstein-Barr virus or an upper respiratory virus or a head injury prior to onset. Second, there has been many secondary cause associated with NDPH such as cerebral venous sinus thrombosis, low or high cerebrospinal fluid pressure syndrome, carotid/vertebral dissection, vasculitis, toxins, congenital abnormalities, metabolic disorders, and neoplasms. The authors supported that there were too many migraine features to fulfill ICHD-II criteria for NDPH, a feature now noted in ICHD-3 which permits TTH and/or migraine symptoms [1, 3, 70–74]. The phenotypic similarities between NPDH and migraine should be noted in the ICHD-3.

### Nummular headache

#### Comments

Nummular headache (NH) is a recently described headache syndrome where continuous or intermittent pain is localized to a coin-shaped region of the skull. NH can be a primary headache disorder or secondary to intracranial or extra cranial pathology. There is a case report in the literature of a four-year-old boy who presented with nummular headache co-localized with a patch of discolored hair and a common etiology is proposed [75].

#### Recommendations

We need more cases of nummular headache in children and adolescents to make good recommendations.

### Hypnic headache

#### Comments

Reports of hypnic headache in children are scarce. The hallmark of the headache is the intensity which rouses from sleep, often at the same time, on a nightly basis. The ICHD-2 age requirement of onset after age 50 years was eliminated in the ICHD-3. A recent literature review of six children with hypnic headache (HH), was used to evaluate the validity of the ICHD-3 criteria in children. There are important differences in children with HH compared to adults which required more data without any limitation of ICHD-3 [3, 73, 74].

#### Recommendations

We need more cases of HH in children and adolescents to make good recommendations.

## Primary headache and comorbidities in children and adolescents

Primary headache is strictly correlated to psychiatric comorbidities, especially in children and adolescents. Previous research found that most frequently described psychiatric comorbidities are anxiety disorders, depression and sleep disorders [76]. Also, children with migraine seem to have lower attachment security than children without the disorder. In a recent study Williams et al. (2017) [77] found a complex pathway from migraine to anxiety symptoms mediated by perceived insecurity of paternal attachment. Furthermore, children with primary headache seem to have a reduced ability to identify and describe feelings (that is called alexithymia). Findings from the study of Cerutti et al. (2016) found that both adolescents and mothers suffering from migraine appear to experience greater level of alexithymia than the control group and the co-occurrence of migraine and alexithymia increases the risk of psychopathology [78].

Thus, it is essential to deepen the presence of comorbidities in children and adolescents with primary headache to choose the correct therapy directed not only to improve headache but also the patient’s quality of life.

## Limitations of this paper

In pediatric medicine, the age of the patient and the education of the parents may affect the reliability and validity of the diagnosis. There may also be differences in diagnostic accuracy by language and culture. This paper is based on the data reported in the literature and the personal experience of pediatric headache specialists. The use of interviews, standardized questionnaires and diaries is another important source of variation in the assessment of headache in children and adolescents. We did not use a common database to calculate the validity, sensitivity, or specificity of the ICHD-3 beta criteria. We are planning to organize a prospective, language-adapted study supported by clinical assessments of video-taped interviews. Furthermore, some of our comments were restricted by a sparsity of data or absence of knowledge on the applicability of specific aspects/points to the pediatric population.

## Implications of the paper

This is the first evaluation of the diagnostic criteria of headache by headache experts from all over the world. All authors based their comments and recommendations on their personal experience, with support from the data in the literature specifically pertaining to the pediatric population, even after the publication of ICHD-3 beta. This paper supports the distinction between pediatric and adult headache. We trust that with the accumulation of data, the next version of the ICHD will include specific subsections with separate definitions/criteria of pediatric headache.

## Conclusions

- Children are not simply small adults; they have distinct biopsychosocial attributes that play a clear role in the pathogenesis and presentation of headache disorders, with important differences from adults.
- Different characteristics of headaches between children, adolescence and adults reflect the degree of brain maturation including myelination, brain plasticity, new synapse formation and synaptic reorganization.
- Different characteristics of headaches between children, adolescence and adults reflect the degree of cognitive development. At around two-six years of age, children may be unable to completely differentiate themselves from their environment, so they explain their experience through cause–effect relations.
  - It is important for physicians to be alert to the specific characteristics of pediatric headache for effective diagnosis and management.
  - The next version of the ICHD should include specific subtopics of pediatric headaches.

## Abbreviations

CH: Cluster headache
CM: Chronic migraine
HC: Hemicrania continua
HH: Hypnic headache
ICDH-3: International classification of headache disorders-3
NDPH: New daily persistent headache
NH: Nummular headache
PEH: Primary exercise headache
PH: Paroxysmal hemicranias
RAP: Recurrent abdominal pain
SUNA: Short-lasting unilateral headache attacks with cranial autonomic symptoms
SUNCT: Short-lasting unilateral neuralgiform headache attacks with conjunctival tearing
TACs: Trigeminal autonomic cephalalgias
TTH: Tension-type headache
